# Microbial modulation of host apoptosis and pyroptosis

**DOI:** 10.3389/fcimb.2014.00083

**Published:** 2014-06-19

**Authors:** Yongqun He, Amal O. Amer

**Affiliations:** ^1^Unit for Laboratory Animal Medicine, Department of Microbiology and Immunology, Center for Computational Medicine and Bioinformatics, Comprehensive Cancer Center, University of Michigan Medical SchoolAnn Arbor, MI, USA; ^2^Department of Microbial Infection and Immunity, Department of Internal Medicine, Center for Microbial Interface Biology, Ohio State UniversityColumbus, OH, USA

**Keywords:** apoptosis, pyroptosis, microbial infection, caspase, inflammasome, *Brucella*, *Legionella pneumophila*, *Mycobacterium tuberculosis*

Apoptosis and pyroptosis are two common programmed cell death types induced by various microbial infections. Apoptosis is non-inflammatory programmed cell death and can be triggered through intrinsic or extrinsic pathways and with or without the contribution of mitochondria. Pyroptosis is an inflammatory cell death and is typically triggered by caspase-1 after its activation by various inflammasomes. Non-canonical caspase-11-mediated pyroptosis has been identified. A NLRP3 (cryopyrin)-dependent but casepase-1-independent proinflammatory necrosis called pyronecrosis (Willingham et al., 2007), and a caspase-2-dependent but caspase-1-independent proinflammatory cell death (Chen et al., 2011) have also been reported. Microbial pathogens are able to modulate host apoptosis, pyroptosis, and inflammasomes through different triggers and pathways. The promotion and inhibition of host cell death vary and depend on the microbe types, virulence, and phenotypes.

In this Special Research Topics issue, recent advances in microbial modulation of host programmed cell death, with a special focus on apoptosis and pyroptosis, were captured in a total of 11 research and review articles. The special issue includes three Original Research Articles, five Review Articles, and three Mini Review Articles. Two articles were published for each of the three pathogens: *Brucella* spp. (Bronner et al., 2013; Pei et al., 2014), *Legionella pneumophila* (Abu Khweek et al., 2013; Casson and Shin, 2013), and *Mycobacterium tuberculosis* (Aguilo et al., 2013; Parandhaman and Narayanan, 2014). Modulation of host immune defenses by *Aeromonas* and *Yersinia* species is introduced in Rosenzweig and Chopra (2013). While (Cunha and Zamboni, 2013) summarizes the subversion of inflammasome activation and pyroptosis by eight pathogenic bacteria, (Malireddi and Kanneganti, 2013) introduces the role of type I interferons in inflammasome activation and cell death induced by microbial infections. The apoptosis-associated uncoupling of bone formation and resorption in osteomyelitis is reviewed in Marriott (2013). The intestinal epithelial cell apoptosis in the setting of altered microbiota with enteral nutrient deprivation is reviewed in Demehri et al. (2013).

*Brucella* causes brucellosis, one of the most common zoonotic diseases in the world in humans and a variety of animal species. The *Brucella*-macrophage interaction is critical to *Brucella* virulence. Virulent smooth *Brucella* strains inhibit macrophage cell death. This is an important strategy employed by several intracellular pathogens to maintain the survival of the eukaryotic cell that represents its niche. Many attenuated rough *Brucella* strains induce macrophage cell death. The Original Research Article (Pei et al., 2014) demonstrates that after smooth *Brucella* invade and replicate inside host macrophages, some smooth bacteria can automatically dissociate into rough mutants that can then cause the macrophage cytotoxicity. The cytotoxicity of infected macrophages is critical for *Brucella* egress and dissemination. The macrophage necrotic cell death also induces inflammatory responses and recruits more macrophages to the infection site.

The rough attenuated *B. abortus* vaccine strain RB51 was found to induce caspase-2-mediated but caspase-1-independent apoptotic and necrotic cell death (Chen and He, 2009). Original Research Article (Bronner et al., 2013) from this special issue further illustrates this mechanism. In RB51-infected macrophages, caspase-2 regulates many genes and several cell death pathways: (i) proapoptotic caspases-3 and -8 activation; (ii) mitochondrial cytochrome *c* release and TNFα production; (iii) caspase-1 and IL-1β production driven by caspase-2-mediated mitochondrial dysfunction. Unlike *S. typhimurium*-induced caspase-1-mediated pyroptosis, RB51-induced pore formation does not contribute to RB51-induced proinflammatory cell death. Therefore, caspase-2 appears to act as a “master regulator” that regulates various genes and pathways and induces a hybrid cell death with features of both apoptosis and pyroptosis. The caspase-2-mediated cell death was also conserved in macrophages treated with cellular stress inducers including etoposide, naphthalene, or anti-Fas (Bronner et al., 2013).

Interesting study by Abu Khweek compared the innate immune response of planktonic and biofilm-derived *L. pneumophila*. *L. pneumophila*, the causative agent of Legionnaire's disease, replicates inside macrophages to establish infection. In the Original Research Article (Abu Khweek et al., 2013), the authors demonstrated that compared to planktonic *L. pneumophila*, biofilm-derived *L. pneumophila* (i) replicate more in murine macrophages, (ii) lacks flagellin expression, (iii) do not activate caspase-1 or -7, (iv) trigger less cell death, and (v) are mostly enclosed in vacuoles that do not fuse with lysosomes. Therefore, biofilm-derived *L. pneumophila* which closely reproduces the natural mode of the bacterial infection in human is able to evade the innate immune response in murine macrophages.

The canonical pyroptosis is triggered by the inflammasome, a multi-protein complex assembled in the cytosol to activate caspase-1. A non-canonical inflammasome activates caspase-11 and also leads to pro-inflammatory cell death (Kayagaki et al., 2011). Independently of the inflammasome, caspase-11 promotes the fusion of the *L. pneumophila*-containing vacuole with the lysosome (Akhter et al., 2012). The diverse roles of caspase-11 and routes of activation are described in the mini-review (Casson and Shin, 2013) *L. pneumophila* triggers canonical caspase-1-dependent inflammasome activation through one of two pathways: (i) Type 4 secretion system (T4SS)-regulated flagellin, NAIP5, and NLRC4; (ii) host ASC and NLRP3, and a *L. pneumophila*-derived unknown signal. Molecular details on caspase-11 activation in *L. pneumophila*-infected macrophages remain unclear. Interestingly, the inflammasome pathway appears to cross talk with and autophagy, another immune response (Casson and Shin, 2013).

*Mycobacterium tuberculosis*, another professional intracellular pathogen in this issue, also manipulates cell death. Conflicting results have been reported to support inhibition or induction of apoptosis as a virulence mechanism employed by mycobacteria. This elegant review article (Aguilo et al., 2013), summarizes the evidences showing that ESX-1-induced apoptosis during mycobacterial infection contributes to bacterial virulence. The ESX-1 secretion system regulates the exportation of ESAT-6, a major virulence factor whose secretion is essential for *M. tuberculosis*-induced apoptosis. ESAT-6 appears to trigger the mitochondrial apoptotic pathway through ER-stress activation. ESX-1 dependent apoptosis supports cell-to-cell colonization and bacterial spread. Highly apoptogenic *M. tuberculosis nuoG* mutant showed higher cell-to-cell spread and increased antigen cross-presentation favoring the host. It is evident that apoptosis may benefit the host or mycobacterial pathogen according to different experimental conditions (Aguilo et al., 2013).

The well rounded report (Parandhaman and Narayanan, 2014) summarizes more than 10 different cell death modalities involving *M. tuberculosis*. The paper also reviews how PknE, one of 11 mycobacterial serine/threonine protein kinases, inhibits apoptosis and benefits the bacterial survival.

This issue also comprises a paper (Rosenzweig and Chopra, 2013) that describes toxins secreted by pathogenic *Yersiniae* and most *Aeromonas* species that modulate infected host cell death. The T3SS effector *Yersinia* outer membrane protein J (YopJ) is an acetyltransferase that disrupts MAPK and NF-κB signaling pathways to favor apoptosis and pyroptosis induction. Similarly, *Aeromonas* hydrophila AexU protein induces apoptosis by targeting NF-κB signaling. Additionally *Aeromonas* includes T2- and T6SS effectors that further modulate host immune responses to promote bacterial virulence (Rosenzweig and Chopra, 2013).

The nice paper by Zamboni's group (Cunha and Zamboni, 2013) first reviews different types of inflammasomes that activate caspase-1 (via NLRC4, AIM2, or NLRP3) or caspase-11 and then lead to pyroptosis. These host inflammasomes and pyroptosis pathways can be targeted by microbial factors released via T3SS/T4SS or other mechanisms in different pathogens. This paper reviews the mechanisms employed by eight bacterial species to evade inflammasome activation and pyroptosis induction. These bacteria include *Chlamydia trachomatis*, *Coxiella burnetii*, *Francisella tularensis*, *Legionella pneumophila, Pseudomonas aeruginosa*, *Shigella flexneri*, *Vibrio parahaemolyticus*, and *Yersinia* spp. (Cunha and Zamboni, 2013).

On the host side, the review by Kanneganti's group (Malireddi and Kanneganti, 2013) in this issue introduces the role of type I interferons in inflammasome activation and cell death during infections of five intracellular, four extracellular bacteria, viruses, and fungi.

How cell death can lead to human disease conditions is well described by the review paper (Marriott, 2013) that focuses on osteomyelitis, a severe infection of bone caused by *S. aureus* and *Salmonella* spp. Osteomyelitis is often associated with bone resorption and progressive inflammatory destruction. In the paper Marriott describes the mechanisms underlying the destruction of bone tissue, with a focus on the apoptosis-associated uncoupling of bone formation and resorption in osteomyelitis. Different microbial virulence factors, host response genes and pathways, and their interactions during the formation of osteomyelitis are introduced.

It has been well established that pathogens modulate apoptosis and pyroptosis, but what about microbiota? The paper (Demehri et al., 2013) in this issue introduces a shift in our understanding of intestinal microbiota such as Gram-negative Proteobacteria after enteral nutrient deprivation. The altered microbiota setting leads to increased intestinal proinflammatory cytokines, decreased epithelial cell proliferation, and increased epithelial cell apoptosis. These eventually cause the loss of epithelial barrier function.

The cover image of this E-book summarizes the key findings reported in the original research, review, or mini-review articles included in this e-book.

As briefly introduced above, this special Research Topic issue covers a broad range of cases and reviews demonstrating the modulation of host cell death pathways by different bacterial pathogens and resident microbiota. While huge progress has been made in the past decades, many challenging questions still remain.

## Conflict of interest statement

The authors declare that the research was conducted in the absence of any commercial or financial relationships that could be construed as a potential conflict of interest.

## References

[B1] Abu Khweek A. Fernandez Davila N. S. Caution K. Akhter A. Abdulrahman B. A. Tazi M. (2013). Biofilm-derived Legionella pneumophila evades the innate immune response in macrophages. Front. Cell. Infect. Microbiol. 3:18 10.3389/fcimb.2013.00018 23750338 PMC3664316

[B2] Aguilo N. Marinova D. Martin C. Pardo J. (2013). ESX-1-induced apoptosis during mycobacterial infection: to be or not to be, that is the question. Front. Cell. Infect. Microbiol. 3:88 10.3389/fcimb.2013.00088 24364000 PMC3850411

[B3] Akhter A. Caution K. Abu Khweek A. Tazi M. Abdulrahman B. A. Abdelaziz D. H. (2012). Caspase-11 promotes the fusion of phagosomes harboring pathogenic bacteria with lysosomes by modulating actin polymerization. Immunity 37, 35–47 10.1016/j.immuni.2012.05.001 22658523 PMC3408798

[B4] Bronner D. O'Riordan M. He Y. (2013). Caspase-2 mediates a *Brucella abortus* RB51-induced hybrid cell death having features of apoptosis and pyroptosis. Front. Cell. Infect. Microbiol. 3:38 10.3389/fcimb.2013.00083 24350060 PMC3842122

[B5] Casson C. N. Shin S. (2013). Inflammasome-mediated cell death in response to bacterial pathogens that access the host cell cytosol: lessons from *legionella pneumophila*. Front. Cell. Infect. Microbiol. 3:111 10.3389/fcimb.2013.00111 24409420 PMC3873505

[B6] Chen F. Ding X. Ding Y. Xiang Z. Li X. Ghosh D. (2011). Proinflammatory caspase-2-mediated macrophage cell death induced by a rough attenuated *Brucella suis* strain. Infect. Immun. 79, 2460–2469 10.1128/IAI.00050-11 21464087 PMC3125819

[B7] Chen F. He Y. (2009). Caspase-2 mediated apoptotic and necrotic murine macrophage cell death induced by rough *Brucella abortus*. PLoS ONE 4:e6830 10.1371/journal.pone.0006830 19714247 PMC2729395

[B8] Cunha L. D. Zamboni D. S. (2013). Subversion of inflammasome activation and pyroptosis by pathogenic bacteria. Front. Cell. Infect. Microbiol. 3:76 10.3389/fcimb.2013.00076 24324933 PMC3840304

[B9] Demehri F. R. Barrett M. Ralls M. W. Miyasaka E. A. Feng Y. Teitelbaum D. H. (2013). Intestinal epithelial cell apoptosis and loss of barrier function in the setting of altered microbiota with enteral nutrient deprivation. Front. Cell. Infect. Microbiol. 3:105 10.3389/fcimb.2013.00105 24392360 PMC3870295

[B10] Kayagaki N. Warming S. Lamkanfi M. Vande Walle L. Louie S. Dong J. (2011). Non-canonical inflammasome activation targets caspase-11. Nature 479, 117–121 10.1038/nature10558 22002608

[B11] Malireddi R. K. Kanneganti T. D. (2013). Role of type I interferons in inflammasome activation, cell death, and disease during microbial infection. Front. Cell. Infect. Microbiol. 3:77 10.3389/fcimb.2013.00077 24273750 PMC3824101

[B12] Marriott I. (2013). Apoptosis-associated uncoupling of bone formation and resorption in osteomyelitis. Front. Cell. Infect. Microbiol. 3:101 10.3389/fcimb.2013.00101 24392356 PMC3867676

[B13] Parandhaman D. K. Narayanan S. (2014). Cell death paradigms in the pathogenesis of *Mycobacterium tuberculosis* infection. Front. Cell. Infect. Microbiol. 4:31 10.3389/fcimb.2014.00031 24634891 PMC3943388

[B14] Pei J. Kahl-McDonagh M. Ficht T. A. (2014). *Brucella* dissociation is essential for macrophage egress and bacterial dissemination. Front. Cell. Infect. Microbiol. 4:23 10.3389/fcimb.2014.00023 24634889 PMC3942807

[B15] Rosenzweig J. A. Chopra A. K. (2013). Modulation of host immune defenses by *Aeromonas* and *Yersinia* species: convergence on toxins secreted by various secretion systems. Front. Cell. Infect. Microbiol. 3:70 10.3389/fcimb.2013.00070 24199174 PMC3812659

[B16] Willingham S. B. Bergstralh D. T. O'Connor W. Morrison A. C. Taxman D. J. Duncan J. A. (2007). Microbial pathogen-induced necrotic cell death mediated by the inflammasome components CIAS1/cryopyrin/NLRP3 and ASC. Cell Host Microbe 2, 147–159 10.1016/j.chom.2007.07.009 18005730 PMC2083260

